# High virulence gene diversity in *Streptococcus pyogenes* isolated in Central Italy

**DOI:** 10.7717/peerj.6613

**Published:** 2019-03-20

**Authors:** Daniela Bencardino, Maria Chiara Di Luca, Dezemona Petrelli, Manuela Prenna, Luca Agostino Vitali

**Affiliations:** 1 School of Biosciences and Veterinary Medicine, University of Camerino, Camerino, Italy; 2 School of Pharmacy, University of Camerino, Camerino, Italy

**Keywords:** FCT, Virulence, GAS, *emm* type

## Abstract

Globally, *Streptococcus pyogenes* poses a continuous burden on human health, causing both self-limiting and life-threatening diseases. Therefore, studying the profile of virulence genes and their combinations is essential to monitor the epidemiology and pathogenic potential of this important species. Thus, the aim of this study was to analyze related genetic features of clinical strains collected in Italy in 2012 in order to obtain a valid picture of their virulence profile that could be compared to similar studies made in other countries approximately in the same period. We conducted *emm* typing and fibronectin-collagen-T antigen (FCT) region typing in 122 *Streptococcus pyogenes* strains. Furthermore, several additional virulence genes were screened by polymerase chain reaction. We found correlations between *emm* types and FCT region profiles. *emm1* strains were mainly associated with FCT2 and FCT6, while *emm89* and *emm12* strains were associated with FCT4. FCT5 was mainly represented in *emm4*, *emm6*, and *emm75* strains. Significantly, we defined subtypes for each FCT type based on the differences in single and double loci compared to the reference scheme used for the classification of the FCT region. In addition, new FCT-region variants with differences in multiple loci were also recorded. Cluster analysis based on virulence gene profiling showed a non-random distribution within each *emm* type. This study added new data to existing studies conducted worldwide and revealed new variability scores in circulating *Streptococcus pyogenes* strains and new assortments in well-established virulence gene signatures.

## Introduction

*Streptococcus pyogenes* (group A Streptococcus, GAS) is an important human pathogen that colonizes the pharynx and the skin, causing an array of diseases ranging from mild sore throat and impetigo to invasive and life-threatening infections (Cunningham, 2000).

Like most pathogens, GAS produces many virulence factors (Cunningham, 2000). Currently, eleven virulence factors have been identified as superantigens: three chromosomally encoded genes (*speG*, *speJ*, and *smeZ*) and eight genes harbored by temperate phages (*speA*, *speC*, *speH*, *speI*, *speK*, *speL*, *speM*, and *ssa*) (Friães et al., 2013). Superantigens are effective exotoxins produced, in addition to GAS, by group C/G *Streptococcus dysgalactiae ssp. equisimilis*, *Staphylococcus aureus*, *Yersinia pseudotuberculosis*, and *Mycoplasma arthritidis* (Barnett et al., 2015). Their pathogenic mechanism involves the ability to bypass the typical antigen presentation process generating an overstimulation of the immune inflammatory cascade of events, eventually leading to a toxic shock syndrome (STSS). STSS and invasive diseases have been frequently found in strains carrying *spe*A and *ssa*, together with *spe*K, and *sme*Z (Commons et al., 2014). Scarlet fever has been correlated with the carriage or acquisition of *ssa*, *spe*A, and *spe*C in different studies (Barnett et al., 2015). A robust correlation has been seen between M18 isolates with *spe*L and *spe*M and acute rheumatic fever (Barnett et al., 2015). Also *spe*K-positive M89 isolates have been associated with the last disease. Two additional virulence factors are encoded by genes located on phages: a phospholipase A_2_ (Sla) and a DNAse (Sdn) (Friães et al., 2013).

SpeB and SpeF, whose genes map instead on the bacterial chromosome and were originally described as exotoxins, are actually a cysteine protease and a DNAse, respectively (Friães et al., 2013). SpeB, is a secreted enzyme possessing a relatively indiscriminant substrate specificity, degrading, on the host side, several substrates such as the extracellular matrix, cytokines, chemokines, complement components, immunoglobulins, and serum protease inhibitors. On the pathogen side, SpeB proteolytic cleavage regulates other streptococcal proteins (Nelson, Garbe & Collin, 2011).

However, the primary virulence factor for GAS remains the major surface M protein that is encoded by the *emm* gene (Metzgar & Zampolli, 2011). It is also the basis for the major typing scheme for GAS, thanks to the considerable sequence variability of the 5′-end of the *emm* genes, for which more than 220 variants have been identified. As such, a large number of functions, and interactions with host molecules, have been ascribed to different M protein variants. Among others, this protein has antiphagocytic properties and elicits a substantial host specific immune response.

*emm* types are associated with specific superantigen profiles, and these associations differ in GAS populations collected from different geographical areas (Commons et al., 2008). It is still unknown whether environmental or biological factors may play a role in these associations.

Additionally, genes encoding several virulence factors involved in GAS pathogenesis are located in the fibronectin-collagen-T antigen (FCT) region (Bessen & Kalia, 2002). The FCT region is highly variable in genetic content and may comprise five to 10 Open Reading Frames (ORFs). In addition, all regions contains either *rofA* or *nra* regulatory genes at one end and genes that encode the major pilin backbone protein (BP, often referred to as FctA), one or two accessory pilin proteins (for example, AP2 or FctB), and at least one sortase.

Different FCT types are associated with different subsets of *emm* types, and a genetic linkage between *emm* type and FCT region profile is associated with GAS tissue specificity (Kratovac et al., 2007).

At last, it is worth mentioning the association between *emm* types and the different recognized arrangements of *emm* and *emm*-like genes within the GAS chromosomal region comprising the *emm* gene itself, named *emm* patterns. Five *emm* patterns, named A–E, have been recognized so far (McGregor et al., 2004). As the first three share basic structural properties and are grouped together, one may consider three main *emm* patterns only, namely A–C, D, and E. Notably, also the structure of the *emm* gene associated to each *emm* pattern group is characteristic (McMillan et al., 2012). GAS strains with different *emm* patterns show a different tissue tropism. Those having patterns A–C are throat specialists; those with pattern D are skin specialists, while pattern-E-positive strains have no obvious tissue preference (Bessen, 2009).

Our study described 122 GAS isolated in 2012 in three regions of central Italy (Marche, Lazio, and Umbria). Our investigation focused on the distribution of virulence factors and their correlation with *emm* types as well as with FCT-region types. The set of genes considered for profiling the population under investigation was modulated on the basis of previous investigations to allow inter-studies comparisons and to increment the knowledge on the epidemiology of GAS populations.

## Methods

### Sample collection and identification

In 2012, 122 GAS isolates were collected from symptomatic patients with pharyngotonsillitis from hospital clinical microbiology laboratories in Roma (Ospedale Pediatrico Bambino Gesù), Perugia (Ospedale S. Maria della Misericordia), and Macerata (Laboratorio di Analisi ASL9). Ethical approval and patient informed consent were not required because the work was on isolates only (no specific sensible data on patients were collected) and entirely conducted between 2011 and 2015 when approval for this kind of studies was not mandatory. Isolates were from throat (*n* = 107), rectal (*n* = 1), and vaginal swabs (*n* = 2), skin and wound (*n* = 6), bronchoalveolar lavage (*n* = 1), and normally sterile fluids (blood and pleural fluid, *n* = 5). Identification of clinical GAS was confirmed in our laboratory by standard procedures such as colony morphology, β-hemolysis on blood agar, serogrouping using a latex agglutination test (Streptococcal Grouping kit; Oxoid, Basingstoke, UK), bacitracin susceptibility test (Oxoid, Basingstoke, UK), and, additionally, by polymerase chain reaction (PCR) detection of *dnaB* (Slinger et al., 2011).

### DNA extraction and *emm* typing

DNA extraction was performed using the GenElute Bacterial Genomic DNA kit (Sigma-Aldrich, St. Louis, MO, USA). *emm* typing was performed according to the Center for Disease Control and Prevention (CDC) guidelines (https://www.cdc.gov/streplab/protocol-emm-type.html). To determine the *emm* type, each sequence was compared to the reference sequences available in the CDC database. The *Streptococcus pyogenes* SF370 was used as the reference strain to check for the general quality of DNA extraction/purification and for PCR conditions.

### Virulence gene profiling

The presence of *speA*, *speB*, *speC*, *speH*, *speI*, *speK*, *speL*, *speM*, *smeZ*, *ssa*, *sdn*, and *sla* virulence genes was tested by PCR using specific primer sequences (Table S1). This set of genes is referred to as non-FCT throughout the text. Briefly, each 25 μL test tube contained one μg of chromosomal DNA, 10 mM Tris–HCl (pH 8.3), 50 mM KCl, 1.5 mM MgCl_2_, 200 μM deoxynucleotide triphosphates (dNTPs), one μM oligonucleotide primers, and 0.5 U Taq polymerase (AmpliTaq Gold; Applied Biosystems, Foster City, CA, USA). The cycling program included an initial denaturation at 95 °C for 2 min, followed by 30 cycles at 95 *°*C for 40 s, at primer-specific annealing temperature for 40 s, at 72 °C for 1 min, and one cycle at 72 °C for 5 min. Various quality controls were applied to uncover potential problems with false positive or false negative results. Controls for the template DNA and components of the PCR mixture included amplification of the *dna*B housekeeping gene (Slinger et al., 2011). Two different operators repeated each PCR independently in different days. Whenever results were discordant, genomic DNA was prepared again from freshly cultured cells and PCR repeated. For the specific case of *spe*B, initial screening gave a high proportion of negative PCR (about 25%). As *spe*B is known to belong to the core genome and found present in almost all surveys of GAS populations so far, an additional set of primers was used to validate the first result (*spe*B-2, Table S1).

As a positive control for each superantigen gene PCR, we used the DNA of an isolate positive to that specific gene. The *Streptococcus pyogenes* SF370 was used as the general reference strain to check for quality of DNA preparations to be used in PCR. In particular, it was used as a positive control for amplification of *spe*B, *emm*, and *dna*B.

### FCT typing

PCR amplification using chromosomal DNA and the general design described in the paragraph “Virulence gene profiling” was performed using 13 different primer pairs targeting various virulence genes within the FCT region (Table S2). The sorted profiles allowed the identification of “FCT-region variants,” based upon but not equivalent to the general scheme by Kratovac et al. (2007) (Table 1). At first, the strains whose profiles were matching one Kratovac’s scheme FCT type were assigned the corresponding type number (one to eight, Table 1), referred to as “reference FCT-region variant,” and designated with the capital letter A after the type number (e.g., 1A, 2A, 3A, and so on). Those strains showing an FCT region pattern matching a Kratovac’s scheme FCT type but for a single PCR product identified a “single FCT-region variant” that was named with the capital letter B after the respective reference FCT type number (e.g., 1B, 2B, 3B, and so on). The same rationale was used to classify double FCT-region variants indicated by the letter C after the corresponding reference FCT type number (e.g., 1C, 2C, 3C, and so on). An example is given by FCT-region variant 5. A total of 21 strains had an FCT-region profile matching that of the Kratovac’s scheme (prtF1+rofA+) and are designated as 5A. Three strains had the profile prtF1+rofA+ and were also positive to the bridge region. This last difference made them a “single FCT-region variant” of type 5, indicated as 5B.

**Table 1:** Patterns of genes or locus defining the FCT-region typing scheme according to Kratovac et al. (2007).

FCT-type^§^	Gene or locus within the FCT region										
	*prt*F1	*cpa*	*prt*F2	*sip*A2	Bridge	*fct*A	*srt*C2	*fct*B	*srt*B	*rof*A	*nra*
1	+	−	−	−	−	−	−	−	+	+	−
2	−	−	−	−	−	−	−	−	+	+	−
3	−	+	+	+	+	+	+	+	−	−	+
4	+	+	+	+	+	+	+	+	+	+	−
5	+	−	−	−	−	−	−	−	−	+	−
6	−	−	−	−	−	−	−	−	−	+	−
7	+	−	+	+	+	+	+	+	+	+	−
8	+	+	+	+	+	+	+	+	+	–	+

**Note:**

§ FCT-type 9 is not listed since it is not discriminated using the Kratovac et al. (2007) PCR typing scheme.

In conclusion, the introduced classification is less stringent and depicts general variation within the classical FCT-type scheme by Kratovac and coll. (Kratovac et al., 2007). For convenience and reference, we have provided the whole raw data set (see the raw data in the Supplemental File).

### Data analysis and statistics

Cluster analysis was done in MEGA v6 (Tamura et al., 2013) and confirmed using Statgraphics Centurion v17.1.08 (Statpoint Technologies Inc., Warrenton, VA, USA). The latter software was used to perform the principal component analysis (PCA) as well as the Chi-square tests when indicated (*p* < 0.05 was considered significant for the rejection of the null hypothesis). Data for PCA were input as a binary matrix of 0 (absence of the gene) and 1 (presence of the gene). Display and annotation of the phylogenetic tree were obtained with Interactive Tree Of Life (iTOL) v3 online tools (Letunic & Bork, 2016).

## Results

### Correlation between non-FCT virulence gene profiles and *emm* types

**Figure 1:**
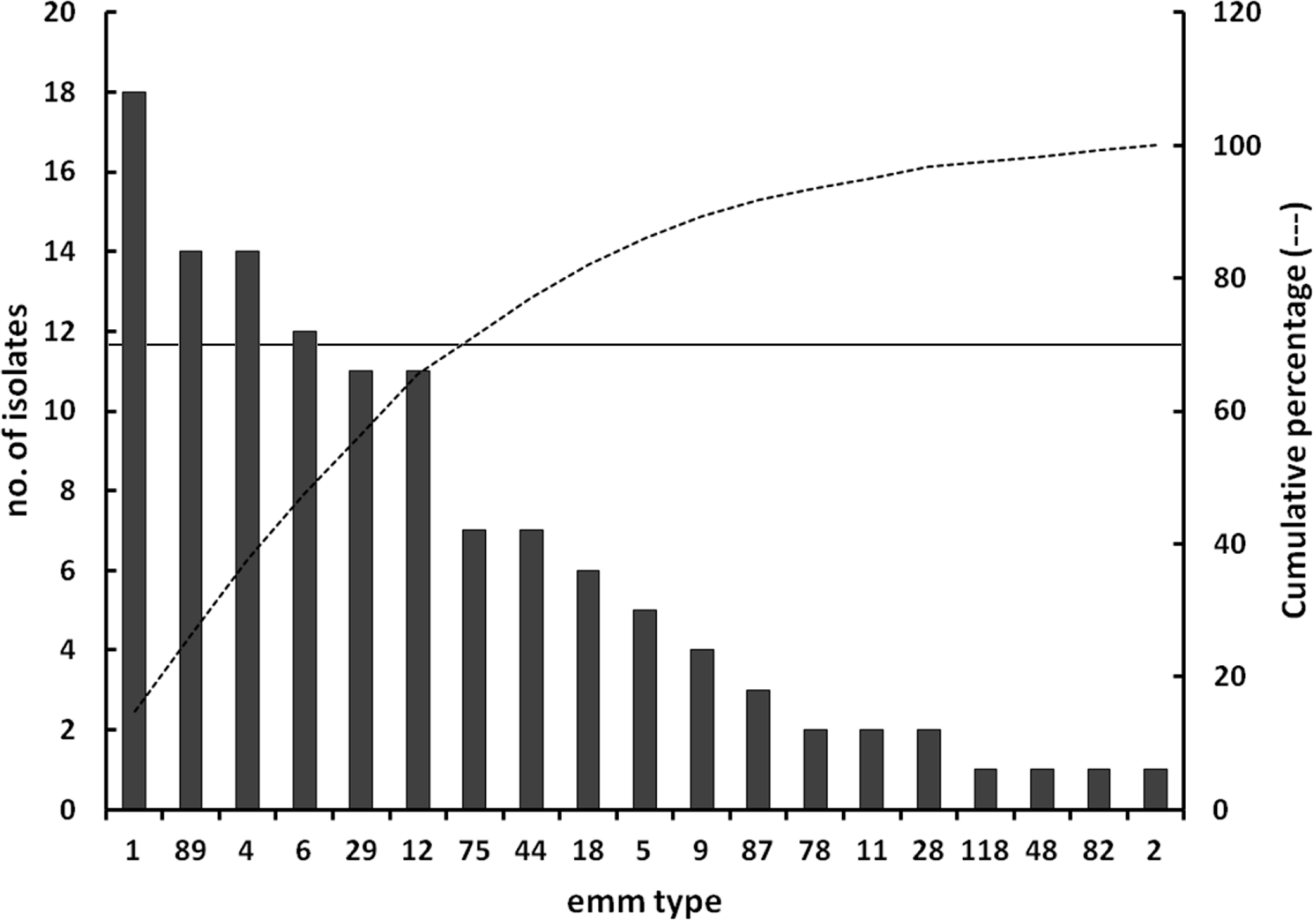
Distribution of *emm* types. Distribution of *emm* types within the *Streptococcus pyogenes* population under investigation. The principal *Y*-axis reports the absolute number of isolates distributing into each *emm* type group, while the secondary *Y*-axis refers to the cumulative function (dotted line) computed from the distribution of *emm* types sorted in a descending order of isolates relative abundance. The continuous horizontal line, referring to the secondary *Y*-axis, indicates the 70% of the study population.

The distribution of *emm* types within the collected strains is reported in Fig. 1. Six *emm* types (1, 4, 89, 6, 12, and 29) accounted for nearly 70% of the population.

**Figure 2:**
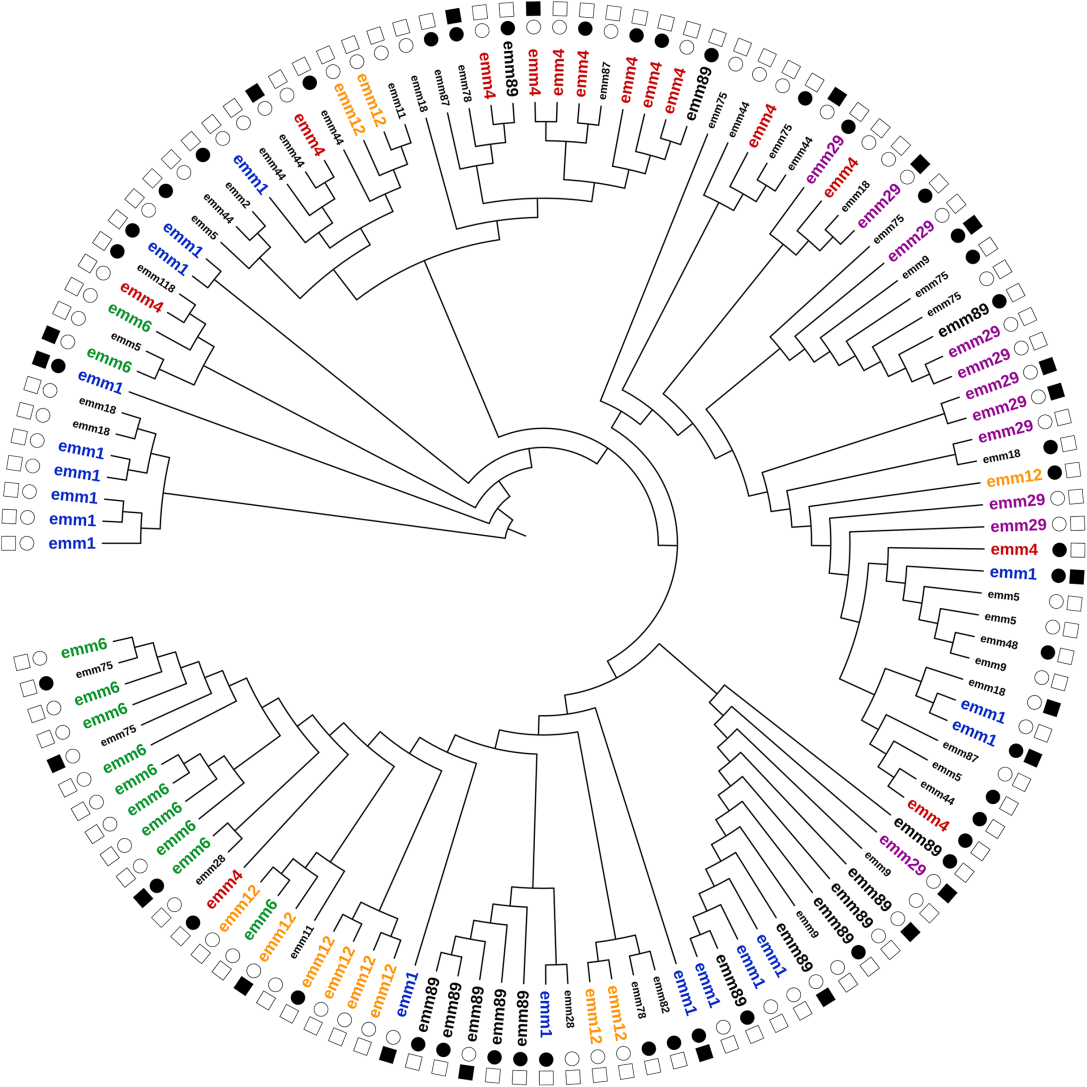
Cluster analysis of *S. pyogenes* strains based on the profile of the entire set of screened virulence genes. For each strain, only the *emm* type (inner circle) and the presence/absence of the genes encoding the two major superantigens, speA and speC (outer circle) are shown. Filled or empty symbols indicate the presence or the absence of the screened gene, respectively. The *emm* types names are in different font size to reflect their relative abundance within the study population.

Although we detected a consistent heterogeneity of non-FCT virulence genes across all *emm* types (Fig. 2), we considered their distribution in the most prevalent *emm* types, namely *emm1*, *emm4*, *emm89*, *emm6*, *emm12*, and *emm29* (Table 2; Fig. 2—labels with a larger font size). *speB* was detected in all strains. The distribution of the other virulence genes in each *emm* type was variable (Fig. 2). For instance, *emm1* strains were present in five distant clusters as well as *emm89* strains (four different clusters). Only six strains were negative to the whole set of virulence genes (excluding *speB*), whereas *speK* and *sla* were the least common (15% and 14%, respectively).

**Table 2:** Distribution of virulence genes within each *emm* type group.

	Total	*emm1*	*emm4*	*emm89*	*emm6*	*emm12*	*emm29*
	*n* = 80	*n* = 18	*n* = 14	*n* = 14	*n* = 12	*n* = 11	*n* = 11
*speC*	30	3	10	7	7	3	0
*speA*	15	9	3	0	3	0	0
*ssa*	16	2	10	2	0	1	1
*sdn*	18	2	8	0	4	1	3
*sla*	11	0	1	0	10	0	0
*speK*	12	1	0	0	7	4	0
*speH*	22	2	2	0	11	7	0
*speI*	14	3	1	0	4	6	0
*speL*	14	0	2	1	0	2	9
*speM*	10	2	2	0	0	2	4
*speB*	80	18	14	14	12	11	11
*smeZ*	22	8	9	1	1	0	3

*speC* was identified in 71% of the *emm4*, 58% of the *emm6*, 50% of *emm89*, 27% of *emm12*, and 17% of the *emm1* strains. Strains positive to *speA* belonged to *emm1* (50%), *emm6* (25%), and *emm4* (21%). The *emm29* strains were the sole type uniformly negative to both *speC* and *speA*.

*sdn* was found in the *emm4* (57%), *emm6* (33%), and *emm1* (11%) strains. *smeZ* was detected in the *emm4* (64%), *emm1* (44%), *emm89* (7%), and *emm6* (8%) strains. We did not observe any *emm89* strain positive to *speA*, *sdn*, *sla*, *speK*, *speH*, *speI*, and *speM*. We did not detect *ssa*, *speL*, and *speM* in the *emm6* strains as well as *sla* and *speL* in the *emm1* strains, whereas in the *emm4* strains only *speK* was not detected (Table 2).

Within *emm1*, *emm4*, *emm6*, and *emm89*, the number of different non-FCT virulence gene profiles was limited (Table S3). The analysis identified 40 genotypes, and the two most common genotypes, G38 and G39, were exclusively found in *emm89* strains (five and six strains, respectively). Similarly, the four G7 strains belonged to *emm1*. Only genotypes G5 and G26 contained three strains. G5 was associated with *emm1*, whereas G26 was associated with *emm4* and *emm89* strains. The rest of genotypes were characterized by one or at least two strains and were not considered for further comparative analysis and discussion.

**Figure 3:**
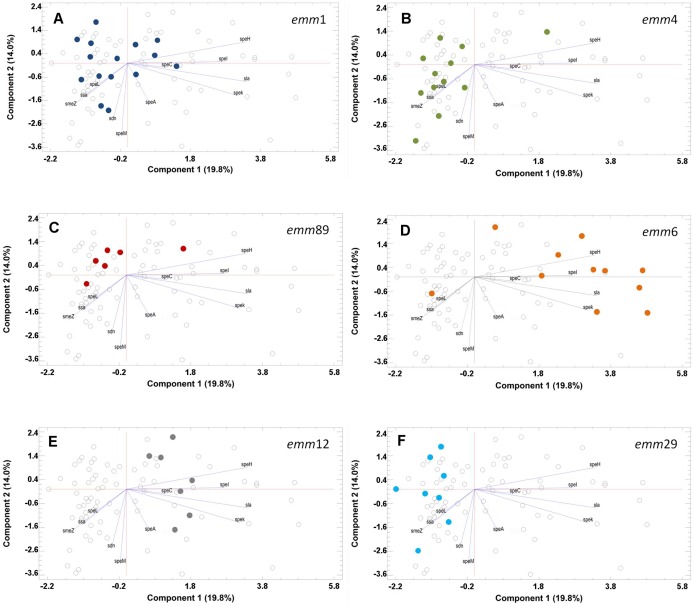
Principal component analysis (PCA) of the contribution of virulence genes to diversity. Only plots referring to the six more prevalent *emm* types in the study population are reported. Each panel shows, as colored dots (different for each *emm* type), the strains sharing at least the same *emm* type (A: *emm1*–dark blue; B: *emm4*–green; C: *emm89*–red; D: *emm6*–orange; E: *emm12*–grey; F: *emm29*–light blue). Strains sharing similar virulence genes’ profiles tend to cluster in the same area of the 2D plot and those having the very same profile spot on the same point within the 2D plot. Hence, the number of spots does not necessarily reflect the number of strains belonging to a definite *emm* type group as per the distribution given in Fig. 1.

Variability in non-FCT virulence genes among *emm1* strains unmasked a substantial heterogeneity wherein none of the sorted principal components accounted for most of the variability (Fig. 3). There were strains containing no virulence genes and groups having *spe*C or *sdn* as the major contributors to variability. Almost the same trend was found in *emm4*, *emm29*, and *emm89* strains. The latter group, however, was composed by strains mostly devoid of virulence genes. The *emm6* strains were characterized by the presence of *speH*/*speI* and *speK*/*sla*. Similarly, the *emm12* strains were diversified by the presence of *speC*.

### FCT typing

A total of 41 (34%) GAS strains showed reference FCT-region variants as previously described (Table 1; Kratovac et al., 2007). The two most frequent were the FCT5 (16%) and FCT4 (10%), which are referred to as FCT5A and FCT4A, respectively. FCT4B was the most prevalent single FCT-region variant (*n* = 18). Compared to the reference FCT-region variant FCT4A, FCT4B lacked *fctA* (*n* = 12) or the bridge region (*n* = 6). Overall, 73 out of 122 strains (60%) showed a single FCT-region variant or a double FCT-region variant of the reference variant. The association of 11 strains (9%) to a specific reference FCT-region variant or a single/double variant was not possible. Their FCT region genetic profile showed extensive differences compared to the reference.

**Figure 4:**
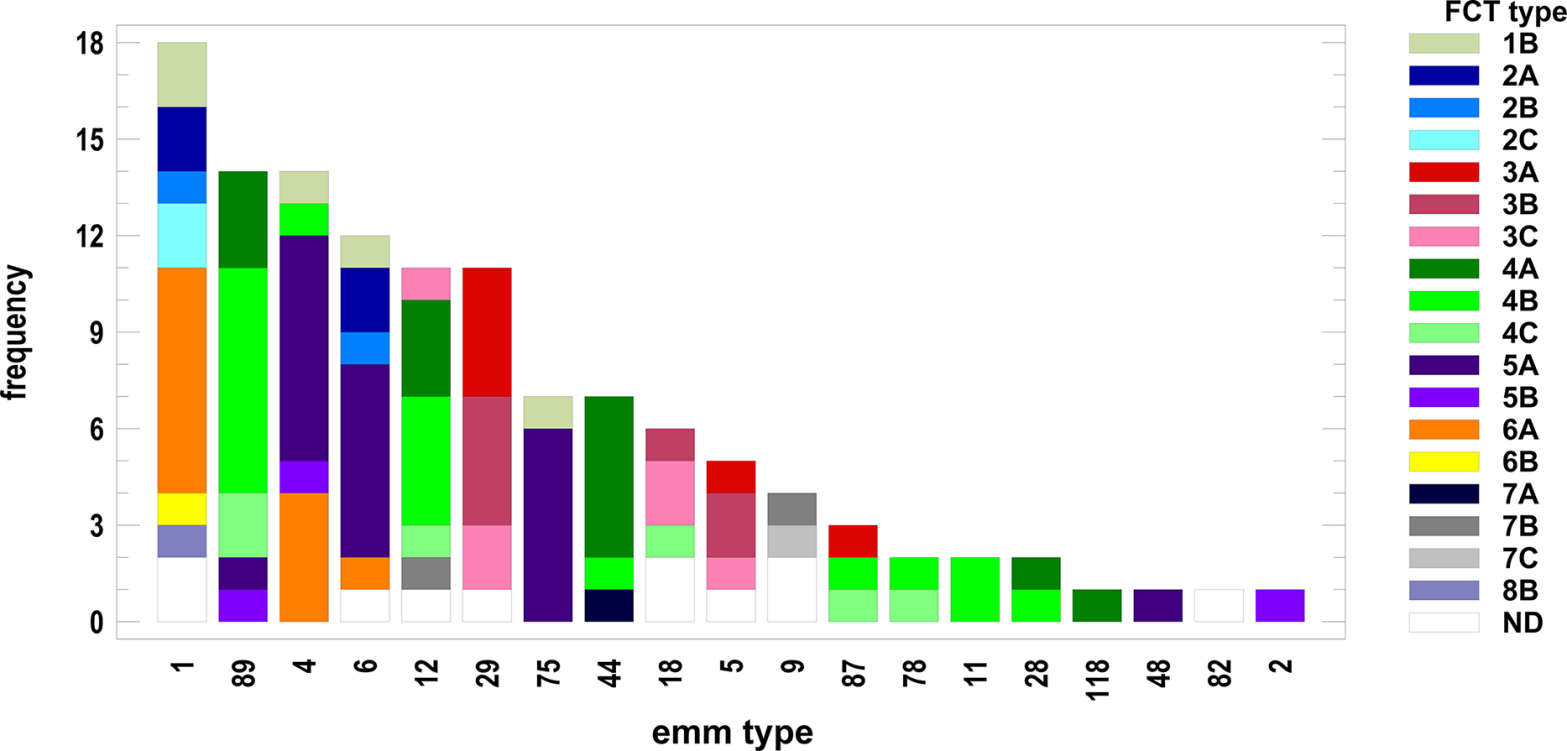
Distribution of fibronectin-collagen-T antigen (FCT) region types variants within each *emm* type group of strains. Frequency on the *Y*-axis refers to the number of strains.

In order to establish a possible association, we cross-tabulated *emm* types and FCT-region variants. We run the test to determine whether or not to reject the hypothesis that the *emm* type and FCT-region variants classifications were independent (Chi-square test). Since the *p*-value was less than 0.001, we rejected the hypothesis that *emm* types and FCT variants were independent at the 99.9% confidence level. Thus, we looked for the associations between FCT-region variants and the most represented *emm* types. As it is shown in Fig. 4, *emm1* strains were mainly associated with FCT regions 2 and 6, while in *emm89* and *emm12* strains the most represented region was FCT4. A good proportion of both *emm4* and *emm6* strains were positive to FCT5. At last, *emm29* strains were almost completely characterized by the FCT3 group of variants.

**Figure 5:**
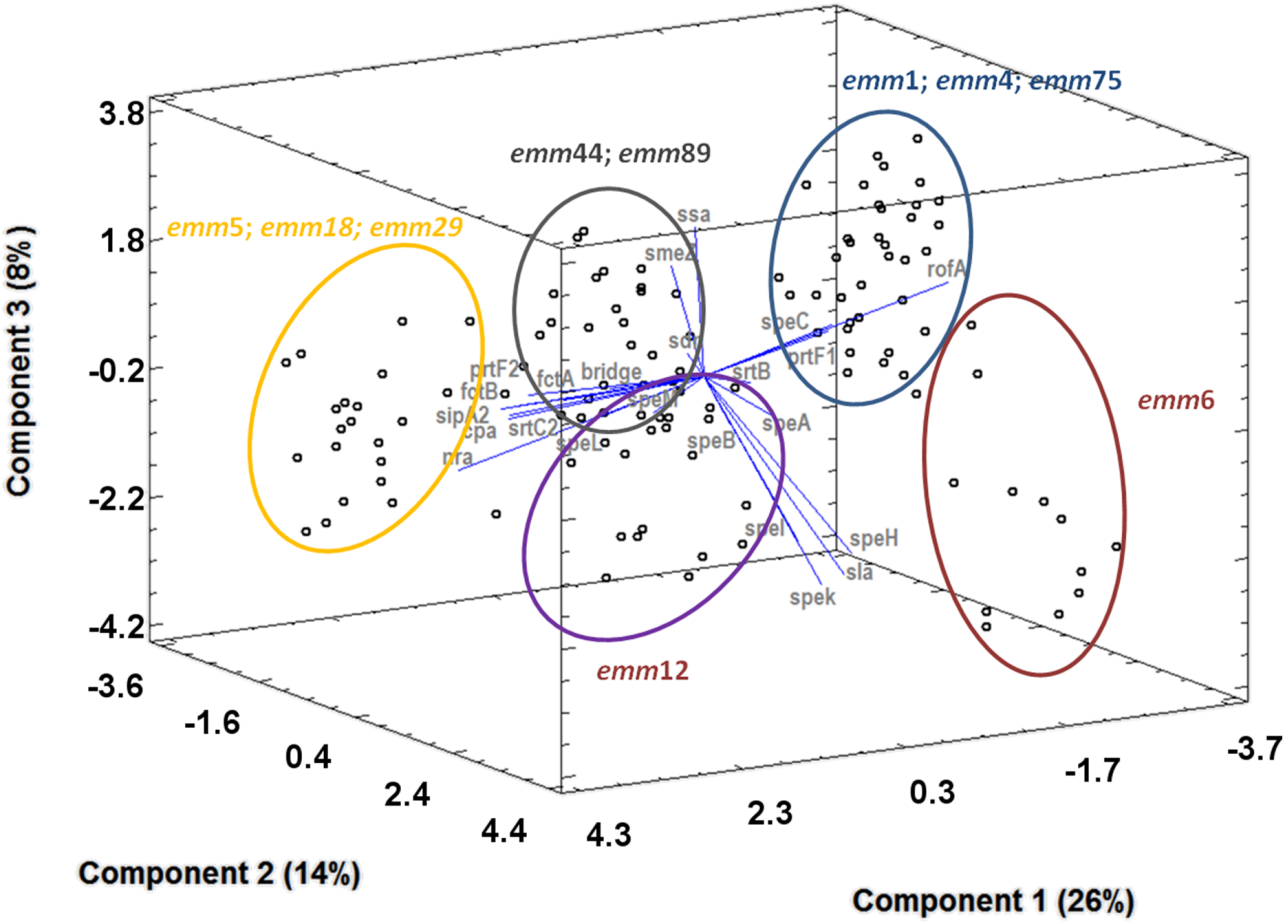
Principal component analysis (3D plot) showing the relative contribution of each virulence gene to the diversity among strains. Clusters of strains (spots) according to their *emm* type is indicated by circles of different colors.

The PCA based on the *emm* type, non-FCT virulence genes, and FCT-region pattern showed an association between *emm* typing, FCT-region variants typing, and virulence gene profiling (Fig. 5). In terms of variability/associations, component 1 was mostly and equally contributed by *fctB* and *prtF2*, followed by *fctA*, *nra*, *srtB*, and *speL. rofA*, *prtF1, srtB*, and *speH*/*I* contributed to variability within component 2, followed by *speK*/*sla* and *speC*. At last, component 3 included *ssa*, *rofA*, *prtF1*, *smeZ,* and *speC*, followed by *srtB*. Based on this variability analysis, same *emm* type strains clustered together. Five major groups were identifiable. The group circled in black (Fig. 5) was mainly composed by *emm44* and *emm89* strains in which there was not a great extent of variability in presence/absence of many genes (proximity to the center of the plot). For instance *prtF1*, *prtF2*, *fctA*, *fctB*, *cpa*, *srtC2* together with *srtB* were overall present, while *ssa* with *speC* contributed to variability within the group as seen by the spread along component 3 axis). The group circled in yellow had *emm* type 5, 18, and 29 strains. They provided a strong contribution to the variability of the component 1 and were positively associated to the presence of *prtF2*, *fctB*, *cpa*, *srtC2*, *sipA2*, *nra*. The opposite could be seen in the case of the cluster circled in blue (*emm1*, *emm4*, *emm75*). Here, the associated genes were mainly *rofA*, *speA*, *smeZ*, and *prtF1*. The two remaining clusters, in violet and red, were made up by strains belonging to *emm12* and *emm6*, respectively. The latter was shifted toward the positive values along the component 2, with a dominant positivity to *srtB*, *speH*/*I, speK*/*sla*, and *speC*. Additionally, *emm* types associable to the same *emm* pattern composed the same cluster. For instance, *emm44* and *emm89* (Fig. 5, black circle) are associated to the *emm* pattern E. *emm5*, *emm18*, and *emm29*, belonging to the *emm* pattern A–C, clustered together (Fig. 5, yellow circle). The cluster made by *emm1*, *emm4*, and *emm75* was an exception (Fig. 5, blue circle), in that *emm1* is usually associated to *emm* pattern A–C, while *emm4* and *emm75* belong to the *emm* pattern E (McMillan et al., 2012).

Overall, when the complete set of virulence genes were considered (i.e., *emm*, non-FCT, and FCT), a total of 83 different profiles were discriminated (Supplemental File), supporting the extremely high genetic heterogeneity of the strains.

### Geographical distribution of *emm* and FCT-region variants

The strains were isolated from three different areas of Central Italy, namely Roma (RM; *n* = 48), Perugia (PG; *n* = 50) and Macerata (MC; *n* = 26). We investigated if there was any association between the isolation area and specific virulence traits. Cross-tabulation of *emm* types by areas and the Chi-square test showed that the hypothesis of independence could be rejected (*p* < 0.001). The measurement of the degree of association between *emm* types and areas showed that there was a 43.1% reduction in error when the *emm* type was used to predict the geographical area where the strain came from. A focus on the six most common *emm* types (1, 4, 89, 6, 12, and 29) showed that the proportions of *emm1* and *emm89* were almost equally represented within the RM, PG, and MC groups. Actually, the RM group had slightly more *emm1* strains than the other two groups, whereas *emm4* strains were scarcely represented in the PG cohort. Conversely, the PG area consistently contributed to the total number of *emm6* and *emm29* strains.

The same analysis was performed to investigate the association between the area of isolation and FCT-region variants typing. The hypothesis of independence for these two variables could be rejected (*p* < 0.01). Given the overall association between the major *emm* types and the FCT-region variants and between *emm* types and geographical area of isolation, a general association between FCT types and geographical area of isolation was expected. In fact, we observed an association between FCT3 variants and the PG area as well as between FCT2/FCT6 variants and the RM area.

## Discussion

Our investigation revealed a high variability of *emm* types and virulence genes among GAS strains from three different areas of Central Italy. Some virulence traits are mostly represented in one area over the other two. This result was not biased by the possible occurrence of major clones circulating in a single area, for instance as a result of epidemics. In fact, the large number of virulence traits considered had a high discriminatory power sorting out 83 different profiles. When compared to the total number of strains (*n* = 122), this number excluded clonality from being a factor influencing clustering and interpretation of variable associations.

Our data confirmed a trend observed in other countries in which *emm1*, *emm4*, *emm89*, and *emm6* (listed in descendent order) were among the prevalent circulating types (Zampaloni et al., 2003; Commons et al., 2008; Steer et al., 2009; Shea et al., 2011), followed by *emm12* and *emm29*. The association between *emm* type and source of isolation was not investigated because the number of strains isolated from throat was highly predominant and would have undermined the significance of the correspondence analysis. Genetic changes in *emm89* strains have recently generated clonal lineages responsible for invasive infections in different parts of the globe (Steer, Danchin & Carapetis, 2007; Beres et al., 2016). The high prevalence of non-invasive *emm89* type strains in our collection confirmed the increasing expansion of this *emm* type that, together with its propensity to switch to more virulent variants, is of concern and claims the continuous monitoring of its diffusion into the population (Latronico et al., 2016). The analysis of the recorded patterns of virulence genes revealed the presence of major clones as already described in previous studies (Vlaminckx et al., 2003; Commons et al., 2008; Friães et al., 2012). We found *emm4* strains with the G25 pattern of virulence genes. They had a wide distribution and were recorded in invasive and non-invasive infections over a long period of time (Commons et al., 2008; Friães et al., 2012). Among the *emm6* strains, the FCT5 and G35 pattern of virulence genes were identified. A similar pattern was present in invasive GAS isolated in The Netherlands from 1992 to 1996 (Vlaminckx et al., 2003). In addition, we described *emm1*/G16 pattern strains that differed for the presence of *sme*Z from *emm1* strains isolated in Portugal (Friães et al., 2012).

Analysis of the results obtained considering the entire set of data and leading to the general picture of Fig. 5 showed different clusters of profiles identified by *emm* types. This observation reinforces the evidence on how *emm* typing continues to represent an exceptional marker of the genetic diversity within a GAS population. The same clustering has been additionally considered in relation to the *emm* patterns, whose general correlation with *emm* types and strains’ source of isolation is well known (Commons et al., 2008; Metzgar & Zampolli, 2011; Friães et al., 2012). Our collection is almost completely made of isolates from cases of pharyngotonsillitis. As a consequence, it is expected to be composed of *emm* types corresponding to patterns A–C (*emm1, 5, 6, 12, 18*) and E (*emm4, 44, 75, 89*). Clusters identified by our total analysis confirmed the *emm* type/*emm* pattern association in all cases but for the one composed by *emm1*, *emm4*, and *emm75* (Fig. 5, blue circle). For instance, *emm1* is usually associated to pattern A–C while the latters to pattern E. This observation clearly indicates that the *emm* type/*emm* pattern association may hinder dissimilarities in important subset of virulence genes, namely superantigens and FCT-region associated ones. As a matter of facts, *emm* types 5, 18, and 29, grouping into one defined cluster by PCA (Fig. 5), were mostly FCT3 and as such positive to the Nra transcriptional regulator gene (Table 1). However, *nra*-positive strains have usually been found associated to *emm* pattern D (skin specialist), while *emm* types 5, 18, and 29 are well known to be associated to *emm* pattern A–C (throat specialist) (Bessen, 2009; McMillan et al., 2012).

## Conclusions

The distribution of *emm* types revealed a complex epidemiological scenario of GAS strains. The detection of 12 virulence genes showed a great degree of clonal heterogeneity within the GAS collection. There was a great structural variability within the FCT region, higher than that previously described (Kratovac et al., 2007), with new FCT-region variants recorded, indicating overall an extremely high degree of genetic shuffling among different parts of the FCT region in GAS. This is most probably related to the evolutionary pressure of the immune system on the different elements exposed on the surface of GAS and encoded by genes mapping within the FCT region such as fibronectin-binding proteins and pili (Mora et al., 2005). Further and continuous molecular epidemiologic studies are needed to increase our understanding of possible associations of virulence determinants and their variants that facilitate host-pathogen interactions. This understanding may help in guiding the design of vaccines against GAS infections.

## Supplemental Information

10.7717/peerj.6613/supp-1 Supplemental Information 1 Set of primers used in PCR screening and distribution of virulence genes.

10.7717/peerj.6613/supp-2 Supplemental Information 2 Complete set of data (strain name, site of isolation, emm type, virulence gene profiling).

## Additional Information and Declarations

### Data Availability

The following information was supplied regarding data availability: The raw data are available in a Supplemental File.
