# Supplementary material for: High virulence gene diversity in Streptococcus pyogenes isolated in Central Italy
Source: PeerJ. 2019 Mar 20;7:e6613. doi: 10.7717/peerj.6613 (PMC6431245; doi:10.7717/peerj.6613)
Supplement: Supplemental Information 1 [file peerj-07-6613-s001.docx]

**Supplementary tables**

**Table S1. Set of primers used for the polymerase chain reaction screening of non-fibronectin-collagen-T antigen (FCT) region-associated loci.**

| **Gene** | **Primer sequence** | **Reference** |
| --- | --- | --- |
| *speA* | 5'-ATGGAAACAATAAAAAG-3'  5'-TTACTTGGTTGTTAGGTAG-3' | (Matsumoto et al., 2003) |
| *speB* | 5'-TTCTAGGATACTCTACCAGC-3'  5'-ATTTGAGCAGTTGCAGTAGC-3' | (Stanley et al., 1996) |
| *speB-2* | 5’-GGATCCCAACCAGTTGTTAAATCTCT-3’ 5’-AACGTTCTAAGGTTTGATGCCTAC AA-3’ | (Chatellier et al., 2000) |
| *speC* | 5'-TCTAGTCCCTTCATTTGGTG-3'  5'-GTAAATTTTTCAACGACACA-3' | (Matsumoto et al., 2003) |
| *speH* | 5'-GTGAATGTCCAGGGAAAGG-3'  5'-GCATGCTATTAAAGTCTCCATTG-3' | (Matsumoto et al., 2003) |
| *speI* | 5'-AATGAAGGTCCGCCATTTTC-3'  5'-TCTCTCTGTCACCATGTCCTG-3' | (Matsumoto et al., 2003) |
| *speK* | 5'-GTGTGTCTAATGCCACCGTCT-3'  5'-GGAACATATATGCTCCTAGAT-3' | (Matsumoto et al., 2003) |
| *speL* | 5'-CAGCACCTTCCTCTTTCTCG-3'  5'-GGAAAAGAGGGACGCAAG-3' | (Matsumoto et al., 2003) |
| *speM* | 5'-GGATGAGTGAATAAATCGGTAA-3'  5'-AGTCGGGACGATGATAA-3' | (Matsumoto et al., 2003) |
| *smeZ* | 5'-CAATAATTTCTCGTCCTGTGTTTGGAT-3'  5'-GATAAGGCGTCATTCCACCATAG-3' | (Lintges et al., 2007) |
| *ssa* | 5'-TGATCAAATATTGCTCCAAGGTG-3'  5'-TCCACAGGTCAGCTTTTACAG-3' | (Suvorov et al., 2009) |
| *sdn* | 5'-AACGTTCAACAGGCGCTTAC-3'  5'-ACCCCATCGGAAGATAAAGC-3' | (Matsumoto et al., 2003) |
| *sla* | 5'-CTCTAATAGCATCGGCTACGC-3'  5'-AATGGAAAATGGCACTGAAAG-3' | (Matsumoto et al., 2003) |

**Table S2. Set of primers used for the polymerase chain reaction screening of fibronectin-collagen-T antigen (FCT) region-associated loci. PCR conditions are reported in** (Kratovac et al., 2007)

| **Gene** | **Primer sequence** | **Fragment**  **(Kb)** |
| --- | --- | --- |
| *prtF1-A* | 5'-TGCGCGGGTTCTATCGGTTTTGGTCAAGTA-3'  5'-AATTAGTTTTYTCARWGCYTCACGCATTAA-3' | 0.55 |
| *prtF1-B* | 5'-TGCGCGGGTTCTATCGGTTTTGGTCAAGTA-3'  5'-CTCCGTCTCACCAGACTCACCCGCTAGAGGTGATTGGTC-3' | 1.2 |
| *cpa-A* | 5' -GGATATGAGATTGCCGAACCTATTACTTTTAAAG-3'  5'-GGAGCCTGTTTATCTTCCATTCGAATAATATCCAC-3' | 0.75 |
| *cpa-B* | 5'-GAAGGTGACTACTCTAAACTTCTAGAGGGAGCAAC-3'  5'-CCAGTTGGTGGGACAAGATCTTTWCGG-3' | 1.3 |
| *prtF2-A* | 5'-GCTGGTGCAACTATGGAGTTGCGTGATTCATCTGGT-3'  5'-CCAGTTGCTGGTAAACTAGTATTACTCTTTGGC-3' | 0.60 |
| *prtF2-B* | 5'-TGCGCGGGTTCTATCGGTTTTGGTCAAGTA-3'  5'-CCCTGGTTATACTGGTTGGAGTCCTTCTCTAG-3' | 1.2 |
| *sipA2* | 5'-GCTTTCATACGGTTAGTACTTAAGATTTCTATTATTGG-3'  5'-CCTCTCACTCTTAATAGAGTTGAGATTTTCCC-3' | 0.46 |
| *fctA* | 5'-AAATTATTACTTGCTACTGCAATCTTAGCAACTGC-3'  5'-CTCCACCAATAGCCACAATGCTAAGAACTGCAAATGGGC-3' | 1.0 |
| *fctB* | 5'-ATGTTATTTTCTGTCGTAATGATATTAACC-3'  5'-CTAGTAACCCCAGTAATACGATACTTAAGATACCC-3' | 1.3 |
| *srtC2* | 5'-GATGACAATTGTACAGGTTATCAATAAAGCC-3'  5'-CTTGAATAGTACCGACAACGATAACACGATTGTCAG-3' | 0.72 |
| *sof* | 5'-GTATAAACTTAGAAAGTTATCTGTAGG-3'  5'-GGCCATAACATCGGCACCTTCGTCAATT-3' | 0.65 |
| *sipA-srtC2* | 5'-GGGAAAATCTCAACTCTATTAAGAGTGAGAGG-3'  5'-GGCTTTATTGATAACCTGTACAATTGTCATC-3' | 1.3 |
| *srtB* | 5'-ATGGAGGAGGTGTGGCAAAAGGCTAAGGCG-3'  5'-CTAAATAATAGCTATAACCACCCCGAAAGCAGC-3' | 0.74 |

**Table S3. Genotypes defined by the profile of non-fibronectin-collagen-T antigen (FCT) region-associated virulence genes and their association with the *emm* type.**

| **Genotype** | **no. of strains** | ***emm1*** | ***emm4*** | ***emm6*** | ***emm89*** | ***speC*** | ***speA*** | ***ssa*** | ***sdn*** | ***sla*** | ***speK*** | ***speH*** | ***speI*** | ***speL*** | ***speM*** | ***speB*** | ***smeZ*** |
| --- | --- | --- | --- | --- | --- | --- | --- | --- | --- | --- | --- | --- | --- | --- | --- | --- | --- |
|  |  |  |  |  |  |  |  |  |  |  |  |  |  |  |  |  |  |
| G1 | 1 | 1 | 0 | 0 | 0 | + | + | − | − | − | − | − | − | − | − | + | + |
| G2 | 1 | 1 | 0 | 0 | 0 | + | + | − | − | − | − | − | − | − | + | + | + |
| G3 | 1 | 1 | 0 | 0 | 0 | − | − | − | − | − | − | − | − | − | − | + | + |
| G4 | 1 | 1 | 0 | 0 | 0 | − | + | + | − | − | − | − | − | − | + | + | − |
| G5 | 3 | 3 | 0 | 0 | 0 | − | − | − | − | − | − | − | − | − | − | + | − |
| G6 | 1 | 1 | 0 | 0 | 0 | − | − | − | − | − | + | − | − | − | − | + | − |
| G7 | 4 | 4 | 0 | 0 | 0 | − | + | − | − | − | − | − | − | − | − | + | − |
| G8 | 1 | 1 | 0 | 0 | 0 | − | − | − | − | − | − | − | − | − | − | + | + |
| G9 | 1 | 1 | 0 | 0 | 0 | − | − | − | + | − | − | − | − | − | − | + | − |
| G10 | 1 | 1 | 0 | 0 | 0 | − | + | − | + | − | − | − | − | − | − | + | + |
| G11 | 1 | 1 | 0 | 0 | 0 | − | − | + | − | − | − | + | − | − | − | + | − |
| G12 | 1 | 1 | 0 | 0 | 0 | − | + | − | − | − | − | − | − | − | − | + | + |
| G13 | 1 | 1 | 0 | 0 | 0 | − | + | − | − | − | − | − | + | − | − | + | + |
| G14 | 1 | 1 | 0 | 0 | 0 | + | − | − | − | − | − | − | + | − | − | + | − |
| G15 | 1 | 0 | 1 | 0 | 0 | − | − | − | + | − | − | − | − | − | − | + | − |
| G16 | 2 | 0 | 2 | 0 | 0 | + | + | + | + | − | − | − | − | − | − | + | + |
| G17 | 1 | 0 | 1 | 0 | 0 | + | − | − | + | − | − | − | − | − | − | + | + |
| G18 | 1 | 0 | 1 | 0 | 0 | − | + | − | − | − | − | − | − | + | − | + | + |
| G19 | 1 | 0 | 1 | 0 | 0 | + | − | + | − | − | − | − | − | − | − | + | − |
| G20 | 1 | 0 | 1 | 0 | 0 | − | − | + | + | − | − | − | − | − | − | + | − |
| G21 | 1 | 0 | 1 | 0 | 0 | − | − | + | + | − | − | − | − | − | + | + | − |
| G22 | 1 | 0 | 1 | 0 | 0 | + | − | + | + | + | − | − | − | − | − | + | + |
| G23 | 1 | 0 | 1 | 0 | 0 | + | − | + | + | − | − | − | − | + | + | + | + |
| G24 | 1 | 0 | 1 | 0 | 0 | + | − | + | − | − | − | + | − | − | − | + | + |
| G25 | 3 | 0 | 2 | 0 | 1 | + | − | + | − | − | − | − | − | − | − | + | + |
| G26 | 1 | 0 | 1 | 0 | 0 | + | − | − | − | − | − | + | + | − | − | + | − |
| G27 | 1 | 0 | 0 | 1 | 0 | + | − | − | − | − | − | + | − | − | − | + | − |
| G28 | 1 | 0 | 0 | 1 | 0 | − | − | − | − | + | + | + | − | − | − | + | − |
| G29 | 1 | 0 | 0 | 1 | 0 | + | − | − | − | + | − | + | + | − | − | + | − |
| G30 | 1 | 0 | 0 | 1 | 0 | + | − | − | − | + | + | + | + | − | − | + | − |
| G31 | 1 | 0 | 0 | 1 | 0 | − | − | − | + | + | − | + | − | − | − | + | − |
| G32 | 1 | 0 | 0 | 1 | 0 | − | − | − | + | − | − | − | − | − | − | + | + |
| G33 | 1 | 0 | 0 | 1 | 0 | + | + | − | + | + | + | + | + | − | − | + | − |
| G34 | 1 | 0 | 0 | 1 | 0 | + | − | − | − | + | − | + | − | − | − | + | − |
| G35 | 2 | 0 | 0 | 2 | 0 | + | − | − | − | + | + | + | − | − | − | + | − |
| G36 | 1 | 0 | 0 | 1 | 0 | − | + | − | − | + | + | + | + | − | − | + | − |
| G37 | 1 | 0 | 0 | 1 | 0 | − | + | − | + | + | + | + | − | − | − | + | − |
| G38 | 5 | 0 | 0 | 0 | 5 | + | − | − | − | − | − | − | − | − | − | + | − |
| G39 | 6 | 0 | 0 | 0 | 6 | − | − | − | − | − | − | − | − | − | − | + | − |
| G40 | 1 | 0 | 0 | 0 | 1 | − | − | − | − | − | − | − | − | + | − | + | − |

**References in Supplementary tables**

Chatellier, S., Ihendyane, N., Kansal, R.G., Khambaty, F., Basma, H., Norrby-Teglund, A., Low, D.E., McGeer, A., Kotb, M., 2000. Genetic relatedness and superantigen expression in group A streptococcus serotype M1 isolates from patients with severe and nonsevere invasive diseases. Infect. Immun. 68, 3523–34. https://doi.org/10.1128/IAI.68.6.3523-3534.

Kratovac Z., Manoharan A., Luo F., Lizano S., Bessen DE. 2007. Population Genetics and Linkage Analysis of Loci within the FCT Region of Streptococcus pyogenes. *Journal of Bacteriology* 189:1299–1310. DOI: 10.1128/JB.01301-06.

Lintges M., Arlt S., Uciechowski P., Plumakers B., Reinert R., Allahham A., Lutticken R., RINK L. 2007. A new closed-tube multiplex real-time PCR to detect eleven superantigens of Streptococcus pyogenes identifies a strain without superantigen activity. *International Journal of Medical Microbiology* 297:471–478. DOI: 10.1016/j.ijmm.2007.03.015.

Matsumoto M., Hoe NP., Liu M., Beres SB., Sylva GL., Brandt CM., Haase G., Musser JM. 2003. Intrahost sequence variation in the streptococcal inhibitor of complement gene in patients with human pharyngitis. *The Journal of infectious diseases* 187:604–612. DOI: 10.1086/367993.

Stanley J., Desai M., Xerry J., Tanna A., Efstration A., George R. 1996. High-Resolution Genotyping Elucidates the Epidemiology of Group A Streptococcus Outbreaks. *Journal of Infectious Diseases* 174:500–506. DOI: 10.1093/infdis/174.3.500.

Suvorov AN., Polyakova EM., McShan WM., Ferretti JJ. 2009. Bacteriophage content of M49 strains of Streptococcus pyogenes. *FEMS microbiology letters* 294:9–15. DOI: 10.1111/j.1574-6968.2009.01538.x.
