# Supplementary material for: High virulence gene diversity in Streptococcus pyogenes isolated in Central Italy
Source: PeerJ. 2019 Mar 20;7:e6613. doi: 10.7717/peerj.6613 (PMC6431245; doi:10.7717/peerj.6613)
Supplement: Supplemental Information 2 [file peerj-07-6613-s002.pdf]

Strain name	Source	*emm*	speC	speA	ssa	sdn
MC001	Oropharyngeal	48	0	0	0	1
MC002	Oropharyngeal	4	0	0	0	1
MC003	Oropharyngeal	89	1	0	0	0
MC004	Oropharyngeal	82	0	0	0	1
MC005	Oropharyngeal	18	0	0	0	0
MC006	Oropharyngeal	9	0	0	0	1
MC007	Oropharyngeal	1	1	1	0	0
MC008	Oropharyngeal	89	0	0	0	0
MC009	Oropharyngeal	44	0	0	1	1
MC010	Oropharyngeal	1	1	1	0	0
MC012	Oropharyngeal	4	1	1	1	1
MC013	Oropharyngeal	4	1	1	1	1
MC014	Oropharyngeal	75	0	1	0	1
MC015	Oropharyngeal	12	0	0	0	0
MC016	Oropharyngeal	1	0	0	0	0
MC017	Oropharyngeal	12	0	0	0	0
MC018	Oropharyngeal	11	1	0	0	0
MC019	Oropharyngeal	12	0	0	1	0
MC020	Oropharyngeal	4	1	0	0	1
MC021	Oropharyngeal	28	1	0	0	0
MC022	Oropharyngeal	44	0	1	1	1
MC023	Oropharyngeal	44	0	0	1	1
MC025	Oropharyngeal	12	0	0	0	0
MC026	Oropharyngeal	44	0	0	1	0
PG001	Oropharyngeal	29	0	0	0	0
PG003	Oropharyngeal	6	1	0	0	0
PG004	Oropharyngeal	75	0	0	0	0
PG005	Vaginal	89	1	0	0	0
PG006	Oropharyngeal	12	0	0	0	0
PG007	Oropharyngeal	118	1	0	0	1
PG008	Blood	6	0	0	0	0
PG009	Oropharyngeal	6	1	0	0	0
PG010	Oropharyngeal	6	1	0	0	0
PG011	Oropharyngeal	75	1	0	0	0
PG012	Oropharyngeal	6	1	0	0	0
PG013	Oropharyngeal	29	0	0	1	0
PG014	Oropharyngeal	29	0	0	0	0
PG017	Oropharyngeal	5	0	0	1	1
PG018	Oropharyngeal	5	0	0	1	0
PG019	Oropharyngeal	2	0	0	1	0
PG020	Bronchoalveolar	29	0	0	0	1
PG021	Oropharyngeal	6	0	0	0	1
PG022	Oropharyngeal	89	0	0	0	0
PG023	Oropharyngeal	1	0	1	1	0
PG024	Oropharyngeal	29	0	0	0	1

PG025	Oropharyngeal	6	0	0	0	1
PG026	Oropharyngeal	75	1	1	0	0
PG027	Oropharyngeal	29	0	0	0	1
PG028	Oropharyngeal	29	0	0	0	0
PG029	Oropharyngeal	89	0	0	0	0
PG030	Skin	29	0	0	0	0
PG031	Oropharyngeal	6	1	1	0	1
PG032	Oropharyngeal	9	0	0	0	0
PG033	Skin	75	0	0	0	0
PG034	Oropharyngeal	29	0	0	0	0
PG036	Oropharyngeal	5	0	0	0	1
PG037	Blood	1	0	0	0	0
PG038	Vaginal	75	0	0	0	0
PG039	Oropharyngeal	9	0	0	0	0
PG040	Oropharyngeal	9	0	0	0	0
PG042	Oropharyngeal	1	0	0	0	0
PG043	Oropharyngeal	5	0	0	0	1
PG044	Oropharyngeal	6	1	0	0	0
PG045	Oropharyngeal	6	1	0	0	0
PG046	Oropharyngeal	1	0	0	0	0
PG047	Oropharyngeal	1	0	1	0	0
PG048	Rectal	89	0	0	0	0
PG049	Wound	89	0	0	0	0
PG051	Oropharyngeal	29	0	0	0	0
PG052	Skin	89	0	0	0	0
PG053	Blood	1	0	0	0	0
PG054	Oropharyngeal	4	0	1	0	0
PG055	Oropharyngeal	44	1	0	1	0
PG056	Wound	18	0	0	0	0
RM001	Oropharyngeal	18	0	0	1	0
RM002	Oropharyngeal	12	0	0	0	0
RM003	Oropharyngeal	4	1	0	1	0
RM004	Oropharyngeal	18	0	1	0	0
RM005	Oropharyngeal	12	0	0	0	0
RM006	Oropharyngeal	4	0	0	1	1
RM007	Oropharyngeal	4	0	0	1	1
RM008	Pleural	1	0	0	0	1
RM009	Oropharyngeal	1	0	1	0	0
RM010	Skin	29	0	0	0	0
RM011	Oropharyngeal	1	0	1	0	1
RM012	Oropharyngeal	6	0	1	0	0
RM013	Oropharyngeal	18	0	1	0	1
RM014	Oropharyngeal	87	0	0	1	1
RM015	Oropharyngeal	4	1	0	1	1
RM016	Oropharyngeal	5	0	0	0	1
RM017	Oropharyngeal	78	0	0	0	0

RM018	Oropharyngeal	6	0	1	0	1
RM019	Oropharyngeal	75	1	0	0	1
RM020	Oropharyngeal	12	1	0	0	1
RM021	Oropharyngeal	4	1	0	1	1
RM022	Oropharyngeal	44	1	0	1	1
RM023	Oropharyngeal	44	0	0	1	0
RM024	Oropharyngeal	18	0	1	0	1
RM025	Oropharyngeal	87	1	0	1	1
RM026	Pleural	1	0	0	1	0
RM027	Oropharyngeal	4	1	0	1	0
RM028	Oropharyngeal	89	1	0	0	0
RM029	Oropharyngeal	11	1	1	0	0
RM030	Oropharyngeal	89	1	0	0	0
RM031	Oropharyngeal	12	0	0	0	0
RM032	Oropharyngeal	1	0	0	0	0
RM033	Oropharyngeal	12	1	0	0	0
RM034	Oropharyngeal	87	1	0	1	0
RM035	Oropharyngeal	89	1	0	0	0
RM036	Oropharyngeal	12	1	0	0	0
RM037	Oropharyngeal	4	1	0	1	0
RM038	Oropharyngeal	4	1	0	1	0
RM039	Oropharyngeal	78	1	0	1	0
RM040	Oropharyngeal	89	0	0	0	0
RM041	Oropharyngeal	1	0	1	0	0
RM042	Oropharyngeal	1	0	1	0	0
RM043	Oropharyngeal	28	1	0	1	0
RM044	Oropharyngeal	1	1	0	0	0
RM045	Oropharyngeal	1	0	1	0	0
RM046	Oropharyngeal	4	1	0	0	0
RM047	Oropharyngeal	89	1	0	1	0
RM048	Oropharyngeal	89	1	0	1	0

sla	spek	speH	speI	speL	speM	speB
0	0	0	0	0	0	1
0	0	0	0	0	0	1
0	0	0	0	0	0	1
0	0	0	1	0	0	1
0	0	0	0	1	0	1
0	0	0	0	0	0	1
0	0	0	0	0	0	1
0	0	0	0	0	0	1
0	0	0	0	0	1	1
0	0	0	0	0	1	1
0	0	0	0	0	0	1
0	0	0	0	0	0	1
1	1	1	1	1	1	1
0	1	0	1	1	0	1
0	0	0	0	0	0	1
0	1	0	1	0	1	1
0	1	0	1	0	1	1
0	1	0	1	0	1	1
0	0	0	0	0	0	1
1	0	1	0	0	0	1
0	0	0	0	0	1	1
0	0	0	0	0	0	1
0	1	0	1	0	0	1
0	0	0	0	0	0	1
0	0	0	0	1	0	1
0	0	1	0	0	0	1
0	0	0	0	1	0	1
0	0	0	0	0	0	1
0	0	1	0	0	0	1
0	0	0	0	0	0	1
1	1	1	0	0	0	1
1	0	1	1	0	0	1
1	1	1	0	0	0	1
0	0	0	0	1	0	1
1	1	1	1	0	0	1
0	0	0	0	1	0	1
0	0	0	0	1	1	1
0	0	0	0	0	0	1
0	0	0	0	0	0	1
0	0	0	0	0	0	1
0	0	0	0	0	1	1
1	0	1	0	0	0	1
0	0	0	0	0	0	1
0	0	0	0	0	1	1
0	0	0	0	1	1	1

0	0	0	0	0	0	1
1	0	1	1	0	0	1
0	0	0	0	1	0	1
0	0	0	0	1	0	1
0	0	0	0	1	0	1
0	0	0	0	1	0	1
1	1	1	1	0	0	1
0	0	0	0	1	0	1
0	0	0	0	1	0	1
0	0	0	0	1	0	1
0	0	0	0	0	0	1
0	0	0	0	0	0	1
0	0	0	0	1	0	1
0	0	0	0	0	0	1
0	0	0	0	0	0	1
0	0	0	0	0	0	1
0	0	0	0	0	0	1
1	0	1	0	0	0	1
1	1	1	0	0	0	1
0	1	0	0	0	0	1
0	0	0	0	0	0	1
0	0	0	0	0	0	1
0	0	0	0	0	0	1
0	0	0	0	1	1	1
0	0	0	0	0	0	1
0	0	0	0	0	0	1
0	0	0	0	1	0	1
0	0	0	0	1	1	1
0	0	0	0	0	1	1
0	0	0	0	0	0	1
0	0	1	1	0	0	1
0	0	0	0	0	0	1
0	0	0	0	0	1	1
0	0	1	1	0	0	1
0	0	0	0	0	0	1
0	0	0	0	0	1	1
0	0	0	0	0	0	1
0	0	0	0	0	0	1
0	0	0	0	0	0	1
0	0	0	0	0	0	1
1	1	1	1	0	0	1
0	0	0	0	0	0	1
1	0	0	0	0	0	1
1	0	0	0	0	0	1
1	0	1	0	0	0	1
0	0	0	1	0	0	1

1	1	1	0	0	0	1
0	1	0	0	1	1	1
0	0	1	0	1	0	1
0	0	0	0	1	1	1
1	1	0	0	1	1	1
1	1	0	0	0	1	1
0	0	0	0	1	1	1
0	0	0	0	0	0	1
0	0	1	0	0	0	1
0	0	1	0	0	0	1
0	0	0	0	0	0	1
0	0	1	0	0	0	1
0	0	0	0	0	0	1
0	0	1	0	0	0	1
0	0	0	0	0	0	1
0	0	1	0	0	0	1
0	0	0	0	0	0	1
0	0	0	0	0	0	1
0	0	1	0	0	0	1
0	0	0	0	0	0	1
0	0	0	0	0	0	1
0	0	0	0	0	0	1
0	0	0	0	0	0	1
0	0	0	0	0	0	1
0	0	0	1	0	0	1
0	0	0	1	0	0	1
0	0	0	1	0	0	1
0	0	1	1	0	0	1
0	0	1	1	0	0	1
0	0	0	0	0	0	1
0	0	0	0	0	0	1

smeZ	prtF1	cpa	prtF2	sipA2	bridge	fctA
0	1	0	0	0	0	0
0	1	0	0	0	0	0
0	1	0	0	0	1	0
0	1	0	1	0	1	0
1	0	1	1	1	1	0
0	1	0	1	1	0	1
1	0	0	0	0	0	0
0	1	1	1	1	1	0
0	1	1	1	1	1	0
1	0	0	0	0	0	0
1	1	0	0	0	0	0
1	1	0	0	0	0	0
0	1	0	0	0	0	0
0	1	1	1	1	1	1
1	0	0	0	0	0	0
0	1	1	1	1	1	1
0	1	1	1	1	0	1
0	1	1	1	1	1	1
1	1	0	0	0	0	0
0	1	1	1	1	1	1
1	1	1	1	1	1	1
0	1	1	1	1	1	1
0	1	0	1	1	1	0
0	1	0	1	1	1	1
0	0	1	1	1	1	1
0	1	0	0	0	0	0
0	1	0	0	0	0	0
0	1	1	0	1	0	1
0	1	1	1	1	1	0
1	1	1	1	1	1	1
0	1	0	0	0	0	0
0	1	0	0	0	0	0
0	1	0	0	0	0	0
0	1	0	0	0	0	0
0	1	0	0	0	0	0
1	0	1	1	1	1	1
0	0	1	1	1	0	0
0	0	1	1	1	1	1
0	0	1	1	1	0	0
0	1	0	0	0	1	0
0	1	1	1	1	0	1
0	1	0	0	0	1	0
0	1	1	1	1	0	0
0	1	0	0	0	1	0
1	0	1	1	1	0	1

1	1	0	0	0	0	0
0	1	0	0	0	1	0
0	0	1	1	1	1	0
0	1	1	1	1	1	1
0	1	1	1	1	1	1
1	0	1	1	1	1	1
0	1	0	1	1	0	0
0	1	0	1	1	0	0
0	1	0	0	0	0	0
0	0	1	1	1	0	1
0	0	1	1	1	1	0
0	0	0	0	0	0	0
0	1	0	0	0	0	0
0	0	0	1	1	0	1
0	1	0	1	1	0	1
0	0	0	0	0	0	0
0	0	1	1	1	1	0
0	0	0	0	0	0	0
0	0	0	0	0	0	0
0	0	0	0	0	0	0
0	0	0	0	1	0	0
0	1	1	1	1	0	1
0	1	1	1	1	0	1
0	0	1	0	1	0	1
0	1	1	1	1	1	1
1	0	0	0	0	0	0
1	0	0	0	0	0	0
0	1	1	1	1	1	1
1	0	1	1	1	0	1
1	0	1	1	1	0	0
0	1	1	1	1	1	0
0	1	0	0	0	0	0
1	0	1	1	1	0	0
0	1	1	1	1	1	0
0	1	0	0	0	0	0
0	1	0	0	0	0	0
0	0	0	0	1	0	0
0	0	0	0	0	0	0
0	0	1	1	1	1	1
1	0	0	0	1	0	0
0	0	0	0	0	0	0
0	0	1	1	1	1	1
0	0	1	1	1	1	1
1	0	0	0	0	0	0
1	0	1	1	1	1	1
0	1	1	1	1	0	0

0	0	0	0	1	0	0
0	1	0	0	0	0	0
0	0	1	1	1	1	1
1	1	0	1	0	0	0
0	1	1	1	1	1	1
0	1	1	1	1	1	1
1	0	1	1	1	1	1
1	1	1	1	1	1	0
0	0	0	0	0	0	0
1	0	0	0	0	0	0
0	1	1	1	1	1	0
0	1	1	1	1	0	1
0	1	1	1	1	1	0
0	1	1	1	0	1	0
0	0	0	0	0	0	0
0	1	1	1	1	1	0
0	0	1	1	1	1	0
0	1	1	1	1	1	0
0	0	1	1	1	1	0
1	1	0	1	0	0	0
1	0	0	0	0	0	0
0	1	1	1	1	0	1
0	1	1	1	1	1	0
1	0	0	1	1	0	0
1	0	0	1	0	0	0
0	1	1	1	1	1	0
0	1	1	1	1	1	0
1	1	0	1	0	0	0
0	1	1	1	1	0	1
1	1	0	0	0	0	0
0	1	1	1	1	1	1

srtC2	fctB	srtB	rofA	nra
0	0	0	1	0
0	0	0	1	0
0	0	0	1	0
1	1	0	1	0
1	1	1	0	1
1	1	1	1	0
0	0	0	1	0
1	1	1	1	0
1	1	1	1	0
0	0	1	1	0
0	0	0	1	0
0	0	0	1	0
0	0	0	1	0
1	1	1	1	0
0	0	0	1	0
1	1	1	1	0
1	1	1	1	0
1	1	1	1	0
0	0	0	1	0
1	1	1	1	0
1	1	1	1	0
1	1	1	1	0
1	1	1	1	0
1	1	1	1	0
1	1	0	0	1
0	0	0	1	0
0	0	0	1	0
1	1	1	1	0
1	1	1	1	0
1	1	1	1	0
0	0	0	1	0
0	0	0	1	0
0	0	0	1	0
0	0	0	1	0
0	0	0	1	0
1	1	0	0	1
1	1	0	0	1
1	1	0	0	1
1	1	0	0	1
0	0	0	1	0
1	1	0	0	1
0	0	1	1	0
1	1	1	1	0
0	0	1	1	0
1	1	0	0	1

0	0	0	1	0
0	0	1	1	0
1	1	0	0	1
1	1	0	0	1
1	1	1	1	0
1	1	0	0	1
0	1	1	1	0
1	1	1	1	0
0	0	0	1	0
1	1	0	0	1
1	1	0	0	1
0	0	0	1	0
0	0	0	1	0
1	0	1	1	0
0	1	0	1	0
0	0	0	1	0
1	1	0	0	1
0	0	1	1	0
0	0	1	1	0
0	0	1	0	1
0	0	1	0	1
1	1	1	1	0
1	1	1	1	0
1	1	1	0	1
1	1	1	1	0
0	0	1	1	0
0	0	0	1	0
1	1	1	1	0
1	1	0	0	1
0	1	0	0	1
1	1	1	1	0
0	0	0	1	0
1	1	0	0	1
1	1	0	0	1
0	0	0	1	0
0	0	0	1	0
0	0	0	1	0
0	0	0	1	0
1	1	0	0	1
0	0	1	1	0
0	0	0	1	0
0	1	0	1	0
1	1	0	0	1
0	0	0	1	0
1	1	0	1	0
1	1	1	1	0

0	0	1	1	0
0	0	0	1	0
1	1	1	1	0
0	0	0	1	0
1	1	1	1	0
1	1	1	1	0
1	1	0	1	0
1	1	1	1	0
0	0	0	1	0
0	0	0	1	0
1	1	1	1	0
1	1	1	1	0
1	1	1	1	0
1	1	1	1	0
0	0	0	1	0
1	1	1	1	0
1	1	1	1	0
1	1	1	1	0
1	1	0	1	0
0	0	1	1	0
0	0	0	1	0
1	1	1	1	0
1	1	1	1	0
0	0	1	1	0
1	0	1	1	0
1	1	1	1	0
1	1	1	0	1
0	0	1	1	0
1	1	1	1	0
0	0	0	1	0
1	1	1	1	0
